# Operational experiences associated with the implementation of near point-of-care early infant diagnosis of HIV in Myanmar: a qualitative study

**DOI:** 10.1186/s12913-021-06797-3

**Published:** 2021-08-23

**Authors:** Win Lei Yee, Hla Htay, Yasmin Mohamed, Claire E. Nightingale, Htay Htay Tin, Win Thein, Latt Latt Kyaw, Win Win Yee, Moe Myat Aye, Steven G. Badman, Andrew J. Vallely, David Anderson, Angela Kelly-Hanku, Stanley Luchters

**Affiliations:** 1 Burnet Institute, Yangon, Myanmar; 2 Burnet Institute, Melbourne, Australia; 3 School of Public Health and Preventive Medicine, Monash University, Melbourne, Australia; 4 Monash Rural Health, Monash University, Bendigo, Australia; 5 National Health Laboratory, Yangon, Myanmar; 6 The Kirby Institute for Infection and Immunity in Society, UNSW Sydney, Sydney, Australia; 7 Sexual and Reproductive Health Unit, Papua New Guinea Institute of Medical Research, Goroka, Papua New Guinea; 8 Department of Population Health, Medical College, Aga Khan University, Nairobi, Kenya; 9 Department of Public Health and Primary Care, Ghent University, Ghent, Belgium

**Keywords:** Point-of-care, Early infant diagnosis, HIV, Viral load, GeneXpert, Prevention of mother-to-child transmission, ART initiation, Implementation science, Myanmar

## Abstract

**Background:**

Timely diagnosis and early initiation of life-saving antiretroviral therapy are critical factors in preventing mortality among HIV-infected infants. However, resource-limited settings experience numerous challenges associated with centralised laboratory-based testing, including low rates of testing, complex sample referral pathways and unacceptably long turnaround times for results. Point-of-care (POC) HIV testing for HIV-exposed infants can enable same-day communication of results and early treatment initiation for HIV-infected infants. However, complex operational issues and service integration can limit utility and must be well understood prior to implementation.

We explored and documented the challenges and enabling factors in implementing the POC Xpert® HIV-1 Qual test (Cepheid, Sunnyvale, CA, USA) for early infant diagnosis (EID) as part of routine services in four public hospitals in Myanmar.

**Methods:**

This sub-study was part of a randomised controlled stepped-wedge trial (Australian and New Zealand Clinical Trials Registry, number 12616000734460) designed to investigate the impact of POC testing for EID in Myanmar and Papua New Guinea. Infants recruited during the intervention phase underwent POC testing at the participating hospitals as part of routine care. Semi-structured interviews with 23 caregivers, 12 healthcare providers and 10 key informants were used to explore experiences of POC-EID testing. The research team and hospital staff documented and discussed implementation challenges throughout the study.

**Results:**

Overall, caregivers and healthcare workers were satisfied with the short turnaround time of the POC test. Occasional delays in POC testing were mostly attributable to late receipt of samples by laboratory technicians and communication constraints among healthcare staff. Hospital staff valued technical assistance from the research group and the National Health Laboratory. Despite staff shortages and infrastructure challenges such as unreliable electricity supply and cramped space, healthcare workers and caregivers found the implementation of the POC test to be feasible at pilot sites.

**Conclusions:**

As plans for national scale-up evolve, there needs to be a continual focus on staff training, communication pathways and infrastructure. Other models of care, such as allowing non-laboratory-trained personnel to perform POC testing, and cost effectiveness should also be evaluated.

**Supplementary Information:**

The online version contains supplementary material available at 10.1186/s12913-021-06797-3.

## Contributions to the literature

- Point-of-care (POC) tests can improve rates of early diagnosis of HIV in infants, but can be challenging to implement in resource-limited settings
- POC implementation studies to date have occurred in high-burden countries with high caseloads of HIV-exposed infants; Myanmar’s facilities have low caseloads so staff are less experienced in testing and referral procedures, presenting new challenges
- This is the first study to show the feasibility of implementation of POC HIV testing for infants in routine healthcare services in a low-caseload environment
- POC HIV early infant diagnosis testing was found to be feasible and acceptable to both caregivers and healthcare workers

## Background

In 2017, an estimated 1.8 million children below 15 years of age were living with HIV globally; 180,000 of whom were newly infected [1]. Without antiretroviral treatment (ART), half of all children with HIV die before they turn 2 years old, with as many as a third dying within the first year of life [2–5]. Early diagnosis of HIV-infected infants and initiation of ART is critical to reducing HIV related morbidity and mortality. Despite World Health Organization (WHO) recommendations that all HIV-exposed infants receive a virological test (DNA or RNA) by the age of 6 weeks (4–6 weeks) [6, 7], in 2016, less than half (43%) of all infants born to HIV-positive mothers worldwide were tested within this timeframe [8]. In Myanmar, only 28% of exposed infants had HIV testing before 2 months of age in 2017 [9, 10].

Myanmar has an estimated HIV prevalence of 0.7% [11]. There were 11,000 children aged 0–14 living with HIV, and less than 500 deaths due to AIDS among children within the same age group [11]. Prevention of mother-to-child transmission of HIV (PMTCT) programs have achieved coverage rates of 78% of HIV-infected pregnant women, although localised evaluations have found that despite 88% of pregnant women being initiated onto a regimen of antiretrovirals, only 59% received ART for more than 8 weeks before delivery and one in eight women did not receive any ART before delivery [12]. According to UNICEF, only about half of the new-born (47%) received nevirapine syrup for prevention of mother-to-child transmission [13], resulting in a vertical transmission rate of about 17% [11]. These figures highlight the critical importance of a functional and effective early infant diagnosis (EID) testing and treatment program in reducing infant deaths. In one study, long test result turnaround times (> 7 weeks) were documented for 36% of babies, with a further 33% never receiving their result, and this was worse for those living further from a centralised laboratory [14]. Another evaluation indicated that only 47% of babies received timely EID (before 8 weeks of age), with 27% experiencing a delay in sample collection, and 26% never getting tested [15].

Many of the challenges associated with the effective delivery of an EID program can be negated by employing point-of-care (POC) technologies. These molecular platforms are designed to be simple and quick to operate in settings with only basic infrastructure and can eliminate sample transport issues and reduce turnaround times, often permitting same-day results. This can enable earlier ART initiation for HIV-positive infants, improved linkage to care and reduced loss to follow-up [16–19]. To date, two POC technologies have received WHO pre-qualification for HIV infant diagnosis, the GeneXpert HIV-1 Qual assay (Cepheid, Sunnyvale, CA, USA) and the Alere q HIV-1/2 Detect (Alere Healthcare, Waltham, Massachusetts, USA). While they are not as simple as lateral-flow HIV antibody diagnostic tests, these small molecular testing platforms can diagnose HIV in less than 2 h, and only require minimally trained personnel, ambient room temperatures, reliable electricity supply, and effective waste disposal systems. Many studies have assessed the feasibility and field performance of these two POC technologies in the EID context, reporting accurate performance compared to gold standard laboratory-based testing, and high acceptability to health services and end users [17, 20–23]. However, operational challenges – including those associated with the physical environment, staff time, supervision and monitoring, engaging the manufacturer, and communicating results – have been observed [17, 18, 20–23].

It is important to note that implementation studies to date have occurred in high-burden countries with significant caseloads of HIV-exposed infants (~ 23%) [17]. This contrasts with Myanmar, where there were an estimated 752 new HIV infections due to mother-to-child transmission in 2017 [13] and most facilities have relatively low caseloads (2–7 cases per month) and thus have less practice in testing procedures and referral pathways. The implementation challenges and efficiencies of POC EID are likely to be significantly different between low and high caseload settings.

Locally informed evidence is essential for key stakeholders and policy makers to inform the effective implementation and wider scale-up of POC testing in the EID program in Myanmar to increase testing rates and improve linkage to care. This is the first study to assess the implementation challenges and enabling factors associated with the introduction of the Xpert HIV-1 Qual test in Myanmar’s public health system.

## Methods

### Study design

This qualitative field evaluation was part of a larger multi-country cluster-randomised pragmatic stepped-wedge trial (Australian and New Zealand Clinical Trials Registry, number 12616000734460), which assessed the effectiveness of implementing the POC Xpert® HIV-1 Qual assay for EID of HIV. In this design, the intervention EID test was introduced at each hospital in a randomised stepwise fashion until all hospitals were providing the same POC service. Before commencing the intervention, each site had a one-month transition period, during which no enrolment occurred and when staff were trained, equipment was installed and participant flow and logistics were organised. The study duration was 19 months, between October 2016 and June 2018, with participants enrolled over a 13-month period.

### Study setting and sites

The study took place at four government hospitals in and around Yangon, the biggest city and former capital of Myanmar. The furthest hospital was located 20 km from the National Health Laboratory (NHL – the reference laboratory). Hospitals were eligible for participation if they: i) offered antenatal HIV screening to pregnant women, (ii) provided services for laboratory-based polymerase chain reaction tests for EID, (iii) had an estimated caseload of at least three HIV-exposed infants attending each month; (iv) had the capacity, or potential capacity, to follow infants for at least 6 months after delivery, and (v) had access to long-term HIV care services, including ART, so that HIV-infected children who met national criteria for treatment during the study would have immediate and accelerated access to treatment.

The participating hospital sites were South Okkalapa Women’s and Children’s Hospital (200 beds), Central Women’s Hospital (500 beds), Thanlyin General Hospital (200 beds) and Thingangyun General Hospital (800 beds). All four hospitals had PMTCT services integrated into routine antenatal care services. Following delivery, paediatric care is continued at the paediatric clinic of each hospital.

### Study participants

#### Caregivers

Most HIV-infected mothers were screened for study eligibility and recruited during their hospital stay following childbirth, and additional recruitment took place with caregivers during the infants’ follow-up visit to the PMTCT clinic. Study staff explained the study to interested caregivers and obtained written informed consent prior to enrolment.

Caregivers 18 years or older with a newborn infant were eligible for participation in the study if: a) the parent/caregiver resided within the site-specific catchment area and was willing to provide reliable contact information; b) infant(s) were born to confirmed HIV-infected mothers and were within the first 28 days of life; c) the parent/caregiver was able and willing to give informed consent for enrolment of their infant in the study, and permitted a member of the research team to remind them to re-attend the clinic.

Near the end of the study, convenience sampling was used to recruit participants who had received both standard care and POC EID tests for qualitative interviews. Caregivers who had different experiences from others in terms of repeated blood withdrawal, testing and delayed receipt of results were also purposely invited to participate in the interviews. In-depth interviews were conducted with a total of 23 caregivers.

#### Healthcare workers and key informants

Hospital-based medico-social workers, laboratory technicians and paediatricians involved in the study implementation were invited to participate in qualitative interviews, and all 12 invitees consented to participate. Ten key informants were also interviewed. These were people who were knowledgeable and influential about EID testing and HIV treatment from the four study hospitals, the National AIDS Program and the NHL.

### Study procedures

#### Standard of care EID testing

We followed national standard operating procedures for participants enrolled during the control and intervention phases. Briefly, six dried blood spots were taken from the heel or big toe of the infant, and in exceptional circumstances from venepuncture. Hospital staff transported the samples, usually the following day or after a few days, to the NHL in Yangon, where they were tested with the Abbott RealTime HIV-1 qualitative assay (Abbott, Abbott Park, Illinois, USA). At the same time, hospital staff collected the printed result forms of previously tested samples from NHL. The results were then given to the caregivers at their next follow-up appointment at the hospital.

#### Intervention phase EID testing

In addition to the standard of care testing described above, participants in the intervention phase were tested with the Xpert HIV-1 Qual assay onsite at the hospital laboratory and results integrated into routine services. For this test, 200 μL of capillary or venous blood was collected into EDTA tubes and a trained laboratory technician from the hospital performed the test on the same day, or the following day, depending on their workload and availability. The paper-based Xpert HIV-1 Qual results were recorded and supplied to the consulting doctors or paediatricians and then provided to the caregivers on the same day or at the scheduled appointment within a week if participants could not wait for the results.

#### Data collection from caregivers and healthcare workers

Researchers who were not involved in the implementation of the trial conducted semi-structured interviews with 23 caregivers (22 mothers and one aunt), 12 healthcare workers and 10 key informants to explore the feasibility and acceptability of the POC testing. The interviews were guided by semi-structured interview guides (see Additional files 1 and 2) developed particularly for this study and took between 45 min to 1 h. The study procedures, risks and benefits were explained to the participants and written informed consent was obtained from all participants.

Implementation challenges were observed, and potential ways to overcome them proposed, and recorded in a spreadsheet by the research coordinator throughout the study period. Emerging challenges and alternative strategies were discussed with the study staff during the fortnightly monitoring visits with the research coordinator in order to refine the testing process.

### Data management and analysis

Recordings of interviews with caregivers and healthcare staff were transcribed and translated into English by the qualitative researchers who conducted the interviews. The research coordinator analysed the translated transcripts, using QSR International’s NVivo 12 qualitative data analysis software (QSR International Pty Ltd., Melbourne, Australia). Both deductive and inductive analysis methods were applied to identify themes within the data. Themes derived from participant responses were cross-checked with the observed data recorded by the research coordinator, and relevant quotes from participants were extracted to support the themes of the observational data and meeting notes.

In 2018, UNICEF outlined the key considerations for introducing new POC diagnostic technologies into national health systems [24]. In analysing the results of our study, we applied these key principles when comparing the benefits and challenges of POC technologies compared with conventional laboratory systems. (outlined in Table 1).

**Table 1:** Summary of benefits and challenges of implementing Xpert®HIV-1 Qual at the hospital setting

	Benefits	Challenges/Limitations
Access	• Specific PMTCT clinic day for HIV-infected mothers • Shorter turnaround time (same-day results in some study sites) • Early receipt of results by patients	• Time constraint with PMTCT clinic opening hours in the afternoon • Communication among multi-level healthcare staff required in the process of testing and result return • Logistical constraint (purchase of Xpert®HIV-1 Qual cartridges)
Efficiency	• Short testing time • Early receipt of results by healthcare workers led to early initiation of ART • A system for communicating EID testing and results to patients by healthcare staff and among healthcare staff	• Low throughput of POC test (two samples can be run at a time although instruments with higher capacity such as four-module device are available • The distributor does not provide technical assistance in some locations
Operability	• Workforce of medico-social workers was advantageous for adherence counselling and patient follow-up • Simple procedures of POC test • Accessible refresher training and technical assistance	• Staff shortages and competing workloads • Infrequent running of POC test • Basic computer literacy
Infrastructure	• Minimal requirement for a system for sample and result transport	• Cramped space for staff and patients • Irregular electricity supply

### Ethics

Ethical approval was obtained from the Ethical Review Committee of the Department of Medical Research (Ethics/DMR/2016/115) in Myanmar. In Australia, ethics approval was obtained from the Alfred Hospital Ethics Committee (Project 500/14).

## Results

This study identified the key factors in the successful implementation of POC testing for EID in four public hospitals in Myanmar. Note that a comparative analysis of cost was beyond the scope of the study. Following the UNICEF comparator framework, we will discuss our results under the following themes: access, efficiency, operability and infrastructure.

### Access

Dedicated PMTCT clinics facilitated access to the Xpert HIV-1 Qual assay. One site offered a dedicated clinic once a week, two sites offered a clinic for HIV-positive mothers once a month, and the fourth site had no set PMTCT clinic dates. More frequent PMTCT clinics improved the efficiency of identification and enrolment of infants requiring HIV testing and provision of test results.

In the intervention phase, laboratory technicians usually performed the POC EID on the same day as the doctor’s consultation, taking about 90 min to produce a result. Caregivers generally waited at the hospital to receive the result on the same day.

*I was told that I would receive the result at 3:30 pm. I am very pleased that my baby was tested and [I] knew the result quickly.* (Mother, lives in Thaketa)

Same-day result delivery was acceptable to most caregivers, particularly considering the travel time experienced by some.

*I came by bus since 9:30 am in the morning. The bus took so long. So, I need to come 3 hours earlier (laughing). When I arrived here it was 12:30 pm.* (Mother, lives in Dagon Satekan)

Results were not always returned on the same day and 39% (9/23) of the participating caregivers did not receive the result on the testing day. Laboratory staff were sometimes unable to test the EID sample upon receipt due to competing urgent tasks or because some PMTCT clinics ran late in the afternoon, with samples being received too close to closing time. To overcome this challenge, the healthcare providers encouraged the caregivers with infants due to have EID testing to arrive at the clinic early on that day so that the laboratory received the samples in time.

We also identified additional challenges associated with the existing EID system. While the integrated healthcare service – made up of paediatricians and an HIV team including a medical officer, nurse, counsellor and laboratory technicians – provided comprehensive care to HIV-infected mothers and their infants, there were instances of delays in timely EID testing (both standard of care and POC) and return of results owing to poor communication within these structures. Healthcare providers were given a chance to discuss these communication gaps during the study staff meetings and the site monitoring visits of the research coordinator and developed communication pathways.

*No, they (HIV team) don’t give the request form to us. If we can’t provide the form, [the laboratory technician] did not accept to do the test. The team did not hand over the information to each other.* (Medico-social worker, male)

### Efficiency

Throughput of the Xpert POC EID test is limited by the number of modules in the platform, which in the case of this study, was two at each site. This reduced the number of tests that could be performed in one afternoon session to a maximum of four and resulted in an inability to return results the same day for some participants.

*“I arrived around one pm at the clinic and had the blood sample taking quickly. The lab will be closed at two pm and there were two patients ahead of me. They asked me to come after three days (to take the result).* (Mother, lives in North Dagon)

Another limitation was that there was only one Cepheid distributor of Xpert cartridges in Myanmar. In one instance, our most distant hospital experienced a breakdown of the machine, which then had to be moved to the research office because the distributor did not provide technical assistance that far outside Yangon. The ordering and receipt of Xpert HIV tests took 2–3 months, and the short expiry date of cartridges (6 months at the time of the study), combined with a low caseload, limited the ability to place large orders. This resulted in frequent orders, requiring significant human resources to place and follow up.

Smaller machine issues could be fixed by trained research staff. Staff involved in the implementation found this to be an effective solution to the issues.

*If we had problem we called to Burnet [research team] and asked how to solve it. Sometimes, the error was encountered while the test is running for about one hour. In that case, Burnet staff came to us immediately and helped us.* (Laboratory technician, female)

### Operability

The hospitals’ laboratory staff varied in qualifications and capabilities. Medical technologists, with a four-year training degree, generally took less time to become accustomed to the POC testing procedures, including quality assurance and maintenance, than the less qualified laboratory technicians with diploma degrees. A lack of experience in using computer technology made some laboratory technicians less confident in handling the Xpert testing device, particularly the linked laptop. This challenge diminished over time with practice and by pairing with other staff familiar with the technology.

*Because I had to use the Xpert machine just once a month, I sometimes forgot the procedure [computer operation] so, I wrote down the operating steps in my notes. Sometimes, I had to call [laboratory coordinator]. I also printed out the test procedure.* (Laboratory technician, female)

Overall, however, both medical technologists and less qualified staff found the test acceptable and easy to use.

*No difficulty. Fine. It is like a routine test for me. Not complicated for me.* (Laboratory technician, female)

*Anyway, the test is simple so that it is feasible. No special training needed for blood sample taking as well as preparation of the sample using the test. The important part is maintenance and care for the machine. The operators should be skilled for it.* (Paediatrician, female)

Our study highlighted the critical importance of selecting the right human resources to support the implementation of POC testing. Medico-social workers, who helped both patient care and the social aspects of caregivers and infants, were essential in minimising loss to follow-up. They also facilitated communication between patients and healthcare staff and played an important role in the coordination of testing.

*I asked the clients to inform me in advance when they are coming for the test after their child turns forty-five days. Then I can confirm with them [HIV team] and the lab person if they are available.* (Medico-social worker, male)

We did note that a shortage of staff, high workloads and complex task-sharing were often associated with unclear responsibilities regarding the POC testing and test result delivery process. Laboratory staff highlighted concerns about human resources in interviews.

*It can be solved with increased human resource. [laughs] My workload is a bit tight with the current setting. Because I have both hospital workload and the test with the study.* (Laboratory technician, female)

### Infrastructure

The availability of the POC device reduced the complex and sometimes ad hoc arrangements that were in place for the delivery of dried blood spots to the NHL and the collection of results.

*[There is] no regular schedule for transporting samples to the National Health Laboratory ... three to four samples were sent together. [There are] no assigned staff for sample transport so it is random and up to the chance of a person going to the [reference] laboratory.* (Laboratory technician, female)

*We just need to put the cartridge and the machine runs itself. In the meantime, we can work for other tasks. When it is ready, we just go to machine and get the result.* (Laboratory technician, female)

The POC device, the computer, and storage for the cartridges require considerable space. This was a major issue at one study site which initially had to set the device up in its lunch room. The research coordinator had several discussions with laboratory staff and a medical superintendent until a proper and fixed place was located for the device in the laboratory.

*It (GeneXpert device) is close to our lunch table in other side of the room, our meal at one side. We had challenge in enough space to put the machine in, as the rooms were a bit small. Now it has moved to another room.* (Laboratory technician, female)

During the implementation of this study, the hospital sites were not required to store more than 20 cartridges onsite, because these could be provided regularly by the study office. This reduced the amount of storage space required at each test site, but this will need consideration if testing is scaled up.

Irregular electricity supply was a problem, and although steps were taken to negate this with the installation of an uninterruptible power source, voltage variation caused problems in some test runs.

*I think it was because of the interrupted power supply. When the QC [Quality Control] was run, the power was off. So, the QC didn’t work well.* (Laboratory technician, female)

## Discussion

This is the first study to investigate the implementation of POC EID testing in routine services at public hospitals in Myanmar. Across these four pilot sites, POC HIV EID testing was found to be feasible and acceptable to both caregivers and healthcare workers. Implementation was not without its challenges; infrastructure issues, the need for continual training and developing an appropriate model of care and communication pathway were highlighted.

Staff found the POC test simple, convenient and easy to use. It circumvented many of the logistical challenges associated with transporting samples and collecting results from the NHL. This finding is comparable to that of a study in Cape Town where the providers considered POC technology was simple and easy [21]. It, however, introduced new challenges associated with clinic time management, with some results not being returned on the same day as the consultation because the blood samples were received too late in the day for processing or the Xpert operator was not available. Approaches such as clear communication channels and specifically scheduling appointments for HIV-exposed infants based on the availability of the Xpert machine, operator and healthcare worker need to be considered and structured accordingly if testing is to be efficient. A study in South Africa about HIV testing at point-of-care found that maintaining relationships among healthcare providers was critical to ensure sufficient stock of equipment and test kits, good communication and to manage high workload [20].

While all staff were trained in the operation of the Xpert device, the low caseload of HIV-exposed infants at each site limited the experience of each operator in conducting the test, and this was further complicated by the need to operate a linked computer. High-quality and clear standard operating procedures, frequent refresher training, and a comprehensive quality system are critical factors in ensuring competence. Similar to the findings of a study in Zimbabwe [25], staff with basic computer literacy quickly gained competency to operate the device. For staff with little computer literacy working in a low-caseload remote setting, a POC device with simpler procedures than the Xpert® HIV-1 Qual, such as the GeneXpert Omni single-module device, is an alternative.

Infrastructure issues, predominantly reliable electricity supply, were evident at these sites, and need considerable thought as the country considers scale-up of decentralised HIV testing. Additionally, space for the machine was a problem at one site in this study, although there were no issues with the storage of test cartridges during the pilot implementation. As McCann et al. [26] recommended to invest in POC EID rather than strengthening the laboratory-based system, if Myanmar is to commit to POC HIV EID testing, further consideration must be given to an appropriate storage space at each site or the development of a hub and spoke delivery system.

Accurate calculations of caseload per clinic and costs data are essential in deciding on how to best distribute resources in Myanmar, as are discussions with other health programs, because the same Xpert machine can be used for HIV viral load, hepatitis C diagnosis and monitoring, human papillomavirus testing and tuberculosis testing and monitoring. As resources, workforce and space are all limiting factors for the provision of care in Myanmar, bringing these programs together and using a hub-and-spoke model for sending samples from sites with low caseload to sites with high caseload where the POC machine is located [27] may add efficiencies to the system in terms of optimising machine use, technical expertise, maintenance and infrastructure support. This will require utilization of the Xpert machine with modules appropriate for the caseload of the health facility in order to avoid delays in result communication.

While our study provides information that is critical to consider as POC EID testing is scaled up, it had some limitations. The hospitals participating in this study were all in the same region as the NHL. Their experiences of sample transfer, results communication and access to technical assistance with the NHL may differ from those of more remote health facilities with greater geographical and transport challenges. Staff who operated the Xpert machine in this study were all trained laboratory personnel, and thus we are unable to extrapolate our results with respect to feasibility to non-laboratory staff. Because this was part of a larger research study, dedicated research staff were available to assist with tracing and following up patients and liaising with the Xpert in-country distributor for machine repair and procurement, resources that will not be available upon national scale-up, suggesting our study overestimates the real-world efficiency of POC testing.

## Conclusion

Despite staff shortages and infrastructure challenges, healthcare workers and caregivers found the implementation of the POC EID test feasible and acceptable. The simplicity of the test required minimal supervision, and access to the NHL was an advantage at the pilot sites. The national scale-up strategy needs a continual focus on staff training, communication pathways and infrastructure. Models of care involving non-laboratory-trained personnel performing POC testing and cost effectiveness of POC EID in low caseload settings should also be evaluated.

## Supplementary Information

**Additional file 1.** Interview guide for caregivers bringing their child for EID results

**Additional file 2.** Interview guide for key informants

## Data Availability

The datasets used and/or analysed during the current study are available from the corresponding author on reasonable request.

## Abbreviations

ART: Antiretroviral treatment
EID: Early infant diagnosis
NHL: National Health Laboratory
PMTCT: Prevention of mother-to-child transmission of HIV
POC: Point-of-care
UNICEF: United National Children’s Fund
WHO: World Health Organization
