# Supplementary material for: Operational experiences associated with the implementation of near point-of-care early infant diagnosis of HIV in Myanmar: a qualitative study
Source: BMC Health Serv Res. 2021 Aug 23;21:863. doi: 10.1186/s12913-021-06797-3 (PMC8383426; doi:10.1186/s12913-021-06797-3)
Supplement: Supplementary file 2 — Additional file 2. Interview guide for key informants [file 12913_2021_6797_MOESM2_ESM.docx]

# AAMI Study

## Interview guide for key informants

**Materials and supplies**

- Consent forms (one copy for participant, one copy for the team)
- Question guide for interviewer
- One digital recording device
- Spare Battery for recording device
- Notebook for note-taking and pens
- Refreshments

**To be completed for each interview:**

| Name of interviewer: |  | Date (dd/mm/yyyy): |  |
| --- | --- | --- | --- |
| Start time: |  | End time: |  |
|  | | | |
| Age: |  | Facility Number: | **\|___\|___\|** |
| Participant ID: | **\|___\|___\|___\|** | Has written informed consent been obtained? | Yes / No |

| **Theme / topic** | **Question** | **Probe** |
| --- | --- | --- |
| Opening questions | 1. Can you tell me about your current role? | *Where do you work?*  *What does your job involve?*  *How long have you been in this role?* |
|  | 1. Can you provide for me an overview of the Early Infant Diagnostic program in PNG / Myanmar? | *What is currently working well?*  *What are some of the challenges with the program?*  *What steps have been taken to try and address these issues?*  *Have these issues always been the case or have they only recently become so? Why/why not?* |
|  | 1. From your point of view what is needed to improve early infant diagnosis of HIV in PNG / Myanmar? | *What has already been done to improve EID services?*  *What are some of the opportunities that could be taken to improve the EID program?* |
| Experiences with the AAMI study | 1. Can you tell me about your experiences with the EID Study? | *How have you found being involved in the study?*  *What has your role been?* |
|  | 1. Can you tell me what you think about the Xpert^®^ HIV-1 qualitative test that was used in the EID study? | *What are some of the benefits of the test?*  *What are some of the challenges with the test?*  *How have caregivers responded to it?*  *How have health workers responded to it?* |
| Feasibility, acceptability and implication for quality of services | 1. Can you tell me what impact you think this test has had on health care provision? | *How has it changed the way mothers and babies are cared for?*  *How has it changed the practices of health care workers?*  *How has it changed the workload of health care workers?*  *How have health workers responded to being able to provide results on the same day?*  *What have some of the challenges been with providing results on the same day?* |
|  | 1. Can you tell me what impact the test has had on starting HIV positive babies on treatment? | *Has the test led to infants starting treatment sooner? Why/why not?*  *What are some of the challenges with starting babies on treatment earlier?*  *Have there been any challenges with accessing treatment on the same day?*  *What are some of the benefits for infants of starting treatment earlier?* |
| Scalability of the integrated services with introduction of the Xpert® test | 1. What role do you think the Xpert^®^ HIV-1 qualitative test has in PNG / Myanmar? | *In what setting do you think the test would be most useful?*  *What are some of the benefits of the test for mothers and babies?*  *What are some of the benefits of the test for health workers?*  *What are some of the benefits of the test for the HIV program as a whole?*  *What are some of the challenges with the test?* |
|  | 1. Can you tell me what you think about scaling up the test for use throughout the country? | *How would this be done?*  *How feasible would this be?*  *What concerns would you have about the scale-up?*  *How could these concerns be addressed?* |
| Closing question | 1. Do you have anything else you would like to add about the study or EID testing services in general? |  |
